# Oxaliplatin-Induced Neuropathy in Colorectal Cancer

**DOI:** 10.1155/2011/201593

**Published:** 2011-12-12

**Authors:** Andrew Weickhardt, Keith Wells, Wells Messersmith

**Affiliations:** Division of Medical Oncology, University of Colorado Cancer Center, Aurora, CO 80045, USA

## Abstract

Oxaliplatin use in palliative and adjuvant treatment of colon cancer is frequently limited by cumulative neurotoxicity, leading to reduced quality of life and decreased dose. The mechanism of this neurotoxicity is unclear, but may relate to neuronal voltage-gated sodium channels involving calcium chelation by a metabolite of the drug. Various preventative measures have been tested to reduce the incidence of neurotoxicity, including calcium and magnesium infusions, dose interruption of the drug, and prophylactic neuromodulatory agents. Despite the promising efficacy of these measures, they are not universally accepted. Less is known about the best way to treat established neurotoxicity, which is permanent in some patients, although venlafaxine has shown promise in small clinical trials. This paper analyzes the extent, cause and risk factors for neuropathy, and the potential preventative and therapeutic treatments for oxaliplatin-induced neuropathy.

## 1. Introduction

Oxaliplatin is a 3rd-generation platinum-based chemotherapeutic, possessing the 1,2 diaminocyclohexane-containing carrier ligand, useful in treating advanced colorectal cancer. It is often used in combination regimens with 5-flourouracil [1], capecitabine [2], or 5-flourouracil/irinotecan [3] for the treatment of colorectal cancer. Oxaliplatin has also been studied in clinical trials for the treatment of other cancers but has found the most success in gastrointestinal neoplasms including gastric, esophageal, and pancreatic cancers [4]. Platinum-based chemotherapeutics have effect via cell phase nonspecific mechanisms causing the formation of cross-linking DNA adducts, leading to strand breaks and inhibition of DNA replication [5].

Oxaliplatin produces common side effects of cytopenias, peripheral neuropathy, diarrhea, and nausea [4]. Oxaliplatin-induced neurotoxicity (OXIN) is a recognized dose-limiting complication [6]. This paper analyzes the extent, cause and risk factors for neuropathy, and the potential preventative and therapeutic treatments for OXIN.

## 2. Extent of Problem

OXIN demonstrates two clinically significant types: acute and chronic. Neither type has its mechanism of action fully elucidated. Acute OXIN usually begins with paresthesias and dysesthesias of the hands and feet, but may include the mouth or throat [7]. Its onset may begin during the initial infusion or up to 1-2 days following the administered dose and is often triggered by cold. Typically, the symptoms will resolve spontaneously within days, but often return upon subsequent oxaliplatin administration. It is associated with additional symptoms of muscular hyperactivity including jaw tightness, cramps, and fasciculations [8]. The proposed mechanism of action of acute OXIN includes altering the current of voltage-gated Na(+) channels in response to oxalate, a metabolic by-product of oxaliplatin [9, 10]. In addition, oxalate may interact indirectly with the voltage-gated Na(+) channels through chelation of calcium (Ca) and magnesium (Mg) [11].

Chronic OXIN, in contrast, is thought to be caused by a dose-dependent accumulation of platinum compounds in the dorsal root ganglia, causing neuronal atrophy and apoptosis. It is primarily a sensory neuropathy affecting the extremities and develops based upon the total cumulative dose of oxaliplatin [12].

There is a wide discrepancy in the literature on how to measure and grade OXIN. The Common Terminology Criteria for Adverse Events (CTCAE) is often used by clinicians to score and monitor OXIN. The grading of peripheral neuropathy from the CTCAE version 4.0 [13] is shown in Table 1. Other rating systems include the Total Neuropathy Score (TNS) [14], the Eastern Cooperative Oncology Group (ECOG) toxicity criteria, the oxaliplatin-specific scale [12], and criteria from individual studies or the World Health Organization [8]. Of these systems, none have been validated for oxaliplatin toxicity scoring. The CTCAE and the ECOG criteria have been compared to the TNS with reasonable validity for all chemotherapeutics [15]. Most clinical trials studying oxaliplatin use the CTCAE or the WHO criteria [2, 16, 17].

## 3. Incidence of Oxaliplatin-Induced Neuropathy

Grade 2 or worse neuropathy occurs in approximately 40–50% of patients receiving oxaliplatin, with grade 3 neuropathy occurring in 10–20% of patients [1, 18–20]. The majority of patients have improvement of their symptoms, but there is still a significant proportion of patients left with some symptoms more than 2 years after completing therapy [1, 20, 21]. In the MOSAIC trial, 44% of patients had grade 2 or grade 3 neurotoxicity at the end of treatment, 6% one year after therapy, and 4% after 18 months [1]. In a European trial of oxaliplatin, 26% of patients with grade 3 neurotoxicity had persistent symptoms after a median followup of 28 months [20]. More than 10% of patients in the NSABP C-07 trial who received oxaliplatin had persistent neurological symptoms 2 years after completing therapy [21]. In a small, 25-patient prospective study specifically evaluating the development of OXIN, 56% of patients developed grade 1-2 OXIN and 8% developed grade 3 OXIN based on a modified TNS scoring [22]. An Australian prospective study of OXIN reported that neuropathy of any grade persisted in 79% of patients, and grade 3 neuropathy persisted in 12% of patients after a median followup of 29 months [23].

### 3.1. Risk Factors

Preexistent symptomatic peripheral neuropathy has served as exclusion criteria from trials of oxaliplatin, and data on exacerbation of underlying neurotoxicity by oxaliplatin is limited. However, diabetes mellitus, which carries a high susceptibility of peripheral sensory neuropathy, does not appear to be associated with increased risk of developing OXIN [24]. Age also has not been shown to be a risk factor for the development of OXIN [25]. It has been hypothesized that genetic differences in the glutathione transferase pathway may lead to higher rates of neurotoxicity, perhaps due to a decreased ability to respond to oxaliplatin-induced oxidative stress [26–28]. However there is conflicting evidence whether ^105^Val polymorphisms of glutathione S-transferase (GSTP1) leads to higher rates of OXIN [29, 30] and there are currently no clinical applications of this basic scientific work. Integrin beta-3 polymorphisms have been shown to be unrelated to the development of OXIN, but may be related to its severity [31]. In a genome-wide analysis of 96 colon cancer patients, a group from South Korea showed a possible connection between OXIN and the DDX18 and NRP2 genes, although the putative mechanism of interaction of these in relation to OXIN is uncertain [32].

### 3.2. Prophylaxis

Two different strategies have been advocated to prevent OXIN: (1) a stop-and-go approach with intermittent oxaliplatin dosing and (2) the concurrent use of neuromodulatory agents which include antidepressants, antiepileptics, or calcium and magnesium infusions.

#### 3.2.1. Stop-and-Go Strategy

Given the reversibility of OXIN in the majority of patients, two separate trials have evaluated stopping oxaliplatin after 6 cycles of therapy, and reintroducing the oxaliplatin after a predefined break while continuing the 5 FU backbone of chemotherapy [33–35]. The OPTIMOX1 trial was a European trial involving 620 patients, which compared continuous FOLFOX-4 regimen (oxaliplatin 85 mg/m^2^) to an intermittent FOLFOX-7 regimen (oxaliplatin 130 mg/m^2^ for 6 cycles with infusional 5 FU/LV then a complete oxaliplatin treatment break) with maintenance 5 FU/LV (12 cycles) followed by reintroduction of FOLFOX-7 (6 cycles) [33]. The incidence of neuropathy was less in the intermittent arm between cycle 7 and 18, but the overall rate of grade 3 neurotoxicity was not significantly different (13% intermittent FOLFOX-7 versus 19% FOLFOX-4, *P* = 0.12). Notably, although 60% of patients in the intermittent arm did not receive further oxaliplatin after their scheduled break from treatment, the response rate, progression-free survival, and overall survival were similar in both arms. The CONcePT trial also compared intermittent to continuous oxaliplatin administration, in a 2 × 2 design additionally randomizing patients to either calcium/magnesium infusion or placebo [35]. This trial used modified FOLFOX-7 (oxaliplatin 85 mg/m^2^) and bevacizumab in both arms, but with alternating blocks of 8 cycles of treatment with oxaliplatin with 8 cycles of biweekly 5 FU/LV/bevacizumab in the intermittent treatment arm. Grade 3 neurotoxicity was significantly reduced in the intermittent oxaliplatin arm versus the continuous oxaliplatin arm (10% versus 24%). Dose delay or dose reduction was significantly less in the intermittent arm compared to the continuous arm (8% versus 22%). Additionally, the intermittent oxaliplatin arm had better time to treatment failure and progression-free survival than the continuous arm.

While the improvement in neurotoxicity is appealing in the stop-and-go approach use of oxaliplatin, the continuation of the 5 FU/LV may be important to maintain efficacy relative to continuous oxaliplatin [36]. The OPTIMOX2 trial compared chemotherapy discontinuation after 6 cycles of FOLFOX-7 with maintenance therapy after 6 cycles of FOLFOX-7, as was used in OPTIMOX1. This approach resulted in inferior duration of disease control and inferior survival, and is not recommended [37]. However, it is important to note that this study was terminated early (and thus analyzed as a randomized phase II), and tumor size was required to surpass baseline measurements prior to reintroduction—an approach which is not used commonly in the clinic. The MACRO trial was a large randomized phase III study that evaluated capecitabine and oxaliplatin (XELOX) with bevacizumab for 6 cycles, followed by maintenance XELOX-bevacizumab or single-agent bevacizumab [38]. This study reported a reduction in grade 3/4 neuropathy from 24% in the continuous XELOX-bevacizumab arm to 7% in the bevacizumab alone arm, with no difference in PFS, OS between the two arms. Therefore stop-and-go oxaliplatin use is as efficacious as continuous oxaliplatin usage when either a 5 FU/LV or bevacizumab backbone is used as maintenance, and results in reduced neurotoxicity. The timing for reintroduction of oxaliplatin is uncertain, and the optimum oxaliplatin-based regimen to use with this approach is also unknown.

#### 3.2.2. Calcium and Magnesium

Calcium and magnesium infusions have been tried as prophylactic therapy for OXIN, on the basis that they may bind to oxalate, and reduce its effect on voltage-gated sodium channels [10, 39]. An encouraging retrospective review [40] of a single institution experience with calcium and magnesium infusions, given before and after oxaliplatin administration, has led to three prospective randomized trials of the strategy. Patients in the CONcePT trial were randomized to either placebo, or Ca/Mg infusion (calcium gluconate 1 g, magnesium sulfate 1 g, in 100 mL of 5% dextrose in water, half an hour prior and half an hour after oxaliplatin administration). Interim analysis of the study data which suggested a decreased response rate in the Ca/Mg arm led to premature closure of the trial [34] after enrolment of only 139 patients. However a subsequent independent review demonstrated that there was no significant difference in response rate or time to treatment failure between the groups. Ca/Mg infusion did not lead to a significant reduction in grade 3/4 neurotoxicity either in the continuous oxaliplatin arm (23% Ca/Mg group versus 24% placebo) or in the intermittent arm (11% Ca/Mg group versus 8% placebo). There was also no difference in the groups between delays and dose reductions. A retrospective review of the effectiveness of Ca/Mg infusions on neuropathy from the CAIRO2 trial also reported no substantial benefit in the reduction of grade 3/4 neuropathy [41].

In contrast the double blinded, placebo-controlled N04C7 trial of patients receiving continuous FOLFOX reported significantly lower grade 2 or worse neurotoxicity in the Ca/Mg arm (22% versus 41%) [18]. In addition, the Ca/Mg group had decreased amounts of muscle cramping, but there was no difference in cold sensitivity. The trial was unfortunately closed after only 102 patients were recruited due to concerns regarding the CONcePT trial's early erroneous assessment of inferior response in the Ca/Mg arm, leading to lack of statistical power to determine if there was a difference in dose delay and dose reduction between the two groups. Preliminary data, taken from an early interim analysis of 52 patients on the blinded, placebo-controlled Neuroxa trial, is also supportive of Ca/Mg infusions [42]. There was a significantly lower frequency of grade 3/4 neurotoxicity in the group receiving Ca/Mg infusions (5% versus 24%), and there was no difference in efficacy between the groups.

An explanation for the possible discrepancy between the insignificant outcomes of Ca/Mg in CONcePT and CAIRO2 compared to the benefit seen in the N04C7 and NEUROXA trial is the number of initial oxaliplatin cycles patients received. Half the patients in the CONcePT trial received stop-and-go therapy with oxaliplatin, and all patients in CAIRO2 had at most 6 cycles of oxaliplatin. These measures by themselves have been shown to reduce neurotoxicity, as demonstrated by the OPTIMOX1 trial [33]. In contrast the N04C7 and Neuroxa trial treated patients with continuous oxaliplatin, suggesting the benefit of Ca/Mg infusions are limited to this treatment strategy. However despite the favorable results from the N04C7 and the NEUROXA trial, there are no current data available on the effect of Ca/Mg infusions on longer-term OXIN [53], and a subsequent further randomized NCCTG trial has begun enrollment to answer this question.

#### 3.2.3. Pharmacological Approaches

A variety of pharmacological approaches to prevent neurotoxicity have been studied, but none have shown sufficient success to use routinely. Tables 2 and 3 summarizes the trials, their interventions, and results.

Glutathione, a tripeptide amino acid, has been shown to act as an antioxidant and prevent oxaliplatin accumulation in nerves [54, 55]. A small 52-patient randomized trial of intravenous reduced dose glutathione given with oxaliplatin reported that the rate of grade 3 neurotoxicity at 8 weeks was significantly less in the group receiving glutathione (0% versus 26%) [43]. There was no difference in chemotherapy response. N-acetylcysteine, which increases glutathione blood concentrations, showed promising benefit in preventing OXIN in a small 14-patient study [44]. Glutamine, another amino acid, has shown promising preventative qualities in a small randomized trial of oral supplementation, but larger confirmatory trials are required [45]. The free radical scavenger amifostine was more effective than glutamine in preventing neurotoxicity when given subcutaneously before oxaliplatin-based chemotherapy [46]. More recently, lipoic acid, an antioxidant, was shown to be no better than placebo in preventing platinum-induced neuropathy in a randomized trial of giving the drug orally during chemotherapy [47].

Carbamazepine, a widely used antiepileptic drug that blocks sodium channels, has also been tried as a preventative measure. However, the drug has a wide range of side effects and low therapeutic index, and a randomized trial of the strategy was ineffective [48]. Gabapentin was also ineffective when tried as a preventative measure in a randomised trial [50]. Oxcarbazepine, an analogue of carbamazepine, was effective in a small randomized trial in preventing OXIN [49], but larger confirmatory trials are required.

Xaliproden is an oral drug that acts through the 5HT1 serotonin receptor and has neurotrophic effects [56]. A large randomized trial of xaliproden with 649 patients receiving FOLFOX-4 chemotherapy reported reduced grade 3 neuropathy in patients taking xaliproden (11% versus 17%), but there was no difference in the percentage of patients with complete recovery after finishing treatment with oxaliplatin (49 versus 47 percent), and the use of the drug for this indication has been abandoned [57].

## 4. Treatment

There are few comparative studies to guide clinicians regarding appropriate treatment of established acute or chronic OXIN. Venlafaxine, an antidepressant that is a serotonin and norepinephrine reuptake inhibitor, may be appropriate for the treatment of acute OXIN [51, 58]. A randomized trial of venlafaxine in patients with acute OXIN receiving FOLFOX chemotherapy for adjuvant or palliative treatment of colon cancer demonstrated a reduction in the proportion of patients suffering from acute OXIN from 31.3% in the placebo arm versus 5.3% in the venlafaxine arm (*P* = 0.03) [58]. Although the trial was small with 54 patients, it also demonstrated in a secondary endpoint that venlafaxine was effective in reducing the incidence of chronic OXIN that was grade 3 or worse from 33% in the placebo arm to 0% in the venlafaxine arm, suggestive that venlafaxine therapy may also be useful as a preventative measure. Amitriptyline use resulted in a nonsignificant trend to improvement in sensory neuropathy in a small, 44-patient randomized trial of cancer patients with chemotherapy-induced neuropathy, of whom 11 patients had oxaliplatin-induced pain [59]. However, a negative randomised trial suggests lamotrigine is ineffective as treatment for chemotherapy-induced neuropathy [52]. Based on individual case reports and small nonrandomized series, other potential therapeutic options for patients, once large confirmatory studies are done, include gabapentin [60] and pregabalin [60, 61].

## 5. Conclusion

OXIN causes significant morbidity and is often the dose-limited factor in treatment of advanced colon cancer. Significant advancement has been made to understand the acute and chronic phases of OXIN, but further research is necessary to develop rational therapeutic options. The current literature in prevention of OXIN is inadequate to guide clinical decision making. Larger future studies are needed to further elucidate the most effective strategies of prevention and treatment of OXIN.

## Figures and Tables

**Table 1:** Common terminology criteria for adverse events version 4.03.

Adverse event	Grade 1	Grade 2	Grade 3	Grade 4	Grade 5
Dysesthesia	Mild sensory alteration	Moderate sensory alteration; limiting instrumental ADL	Severe sensory alteration; limiting self-care ADL	—	—
Paresthesia	Mild symptoms	Moderate symptoms; limiting instrumental ADL	Severe symptoms; limiting self care ADL	—	—
Peripheral sensory neuropathy	Asymptomatic; loss of deep tendon reflexes or paresthesia	Moderate symptoms; limiting instrumental ADL	Severe symptoms; limiting self care ADL	Life-threatening consequences; urgent intervention indicated	Death
Peripheral motor neuropathy	Asymptomatic; clinical or diagnostic observations only; intervention not indicated	Moderate symptoms; limiting instrumental ADL	Severe symptoms; limiting self care ADL; assistive device indicated	Life-threatening consequences; urgent intervention indicated	Death

**Table 2:** Prevention of oxaliplatin-induced peripheral neuropathy.

Study reference	Intervention	Results
OPTIMOX1 [33]	Continuous FOLFOX-4 versus intermittent FOLFOX-7	No significant reduction in grade 3 neuropathy in the intermittent arm
CONcePT [34]	Continuous oxaliplatin versus intermittent oxaliplatin	Reduced grade 3 neuropathy in the intermittent arm
	Ca/Mg versus placebo	No reduction in grade 3/4 neuropathy in Ca/Mg group
Gamelin et al. 2004 [40]	Ca and Mg versus historic control	Reduced grade 3 neuropathy in Ca/Mg group
CAIRO2 trial [41]	Ca/Mg versus no prevention treatment	No reduction in grade 3/4 neuropathy in Ca/Mg group
N04C7 trial [18]	Ca/Mg versus placebo	Reduced grade 2 or worse neuropathy in Ca/Mg group
Neuroxa trial (ongoing) [42]	Ca/Mg versus no prevention treatment	Reduced grade 3 neuropathy in Ca/Mg group
Cascinu et al. 2002 [43]	Glutathione versus placebo	Reduced grade 3 neuropathy with glutathione
Lin et al. 2006 [44]	N-acetylcysteine versus placebo	Reduced grades 2–4 neuropathy with N-acetylcysteine
Wang et al. 2007 [45]	Glutamine versus no treatment	Reduced grade 3 neuropathy with glutamine
Lu et al. 2008 [46]	Amifostine versus glutamine	Reduced grades 1-2 and 3-4 in amifostine group
Guo et al. 2011 [47]	Oral lipoic acid versus placebo	No statistical difference in treatment groups
Von Delius et al. 2007 [48]	Carbamazepine versus no prevention treatment	No statistical difference in treatment groups
Argyriou et al. 2006 [49]	Oxcarbazepine versus no prevention treatment	Reduction in incidence of neuropathy in oxcarbazepine group
Mitchell et al. 2006 [50]	Gabapentin versus no prevention treatment	No reduction in neuropathy
Durand et al. 2003 [51]	Venlafaxine versus placebo	Reduced grade 3 neuropathy
Cassidy et al. 2008 [16]	Xaliproden versus placebo	Reduced grade 3 during treatment, but no difference after finishing treatment

**Table 3:** Treatment of oxaliplatin-induced peripheral neuropathy.

Study	Intervention	Results
Durand et al. 2003 [51]	Venlafaxine versus placebo	Reduced grade 3 neuropathy
Rao et al. 2008 [52]	Lamotrigine versus placebo	No reduction in neuropathy
